# Metabolic engineering of *Zymomonas mobilis* for 2,3-butanediol production from lignocellulosic biomass sugars

**DOI:** 10.1186/s13068-016-0606-y

**Published:** 2016-09-02

**Authors:** Shihui Yang, Ali Mohagheghi, Mary Ann Franden, Yat-Chen Chou, Xiaowen Chen, Nancy Dowe, Michael E. Himmel, Min Zhang

**Affiliations:** 1 National Bioenergy Center, National Renewable Energy Laboratory, Golden, 80401 USA; 2 Biosciences Center, National Renewable Energy Laboratory, Golden, CO 80401 USA; 3 Hubei Collaborative Innovation Center for Green Transformation of Bio-resources, Hubei Key Laboratory of Industrial Biotechnology, College of Life Sciences, Hubei University, Wuhan, 430062 China

**Keywords:** *Zymomonas mobilis*, 2,3-Butanediol, Metabolic engineering, Omics, Redox balance, Advanced biofuel, Fermentation, Respiration chain

## Abstract

**Background:**

To develop pathways for advanced biofuel production, and to understand the impact of host metabolism and environmental conditions on heterologous pathway engineering for economic advanced biofuels production from biomass, we seek to redirect the carbon flow of the model ethanologen *Zymomonas mobilis* to produce desirable hydrocarbon intermediate 2,3-butanediol (2,3-BDO). 2,3-BDO is a bulk chemical building block, and can be upgraded in high yields to gasoline, diesel, and jet fuel.

**Results:**

2,3-BDO biosynthesis pathways from various bacterial species were examined, which include three genes encoding acetolactate synthase, acetolactate decarboxylase, and butanediol dehydrogenase. Bioinformatics analysis was carried out to pinpoint potential bottlenecks for high 2,3-BDO production. Different combinations of 2,3-BDO biosynthesis metabolic pathways using genes from different bacterial species have been constructed. Our results demonstrated that carbon flux can be deviated from ethanol production into 2,3-BDO biosynthesis, and all three heterologous genes are essential to efficiently redirect pyruvate from ethanol production for high 2,3-BDO production in *Z. mobilis*. The down-selection of best gene combinations up to now enabled *Z. mobilis* to reach the 2,3-BDO production of more than 10 g/L from glucose and xylose, as well as mixed C6/C5 sugar streams derived from the deacetylation and mechanical refining process.

**Conclusions:**

This study confirms the value of integrating bioinformatics analysis and systems biology data during metabolic engineering endeavors, provides guidance for value-added chemical production in *Z. mobilis*, and reveals the interactions between host metabolism, oxygen levels, and a heterologous 2,3-BDO biosynthesis pathway. Taken together, this work provides guidance for future metabolic engineering efforts aimed at boosting 2,3-BDO titer anaerobically.

**Electronic supplementary material:**

The online version of this article (doi:10.1186/s13068-016-0606-y) contains supplementary material, which is available to authorized users.

## Background

With the thriving interest today in sustainable and secure domestic advanced hydrocarbon production, extensive research has been conducted on microorganisms that naturally produce high titers of intermediates for drop-in fuels upgrading. A number of microorganisms have been recently engineered to produce hydrocarbon or hydrocarbon intermediates. However, there are only a few reports about the development of commercially viable strains for the production of advanced hydrocarbon or hydrocarbon intermediates. 2,3-butanediol (2,3-BDO) is an economically important platform bulk chemical that can be used in a variety of chemical feedstocks, liquid fuels, and biosynthetic building blocks, such as synthetic rubber, solvents, and food additives. For example, dehydration can convert 2,3-BDO into methyl ethyl ketone, which can be used as a liquid fuel additive; and 2,3-BDO can also be deoxydehydrated into 1,3-butadiene, which is a critical building block for synthetic rubber. 1,3-butadiene can also be oligomerized in high yields to gasoline, diesel, and jet fuel [1–11]. 2,3-BDO can be produced efficiently by fermentation with microorganisms, such as *Klebsiella* sp., *Enterobacter* sp., *Serratia* sp., *Bacillus* sp., and the yeast, *Saccharomyces cerevisiae* [1–5, 12–16]. The 2,3-BDO biosynthesis pathway in these microorganisms utilizes three enzymes: acetolactate synthase (Als), acetolactate decarboxylase (AldC), and butanediol dehydrogenase (Bdh), which convert, sequentially, pyruvate to α-acetolactate, acetoin, and then 2,3-butanediol. High 2,3-BDO titer has been reported in native and engineered microorganisms, especially under fed-batch fermentation conditions. This body of work has been reported and reviewed extensively with excellent summaries on 2,3-BDO production pathways, microorganisms, pathway engineering strategies, operation conditions, and downstream processing [1–4]. For example, microbial 2,3-BDO production using different bacterial species, substrates, and fermentation methods has been summarized by Ji et al. [3]. Work to demonstrate heterologous 2,3-BDO production in the heterologous hosts *E. coli* [17–21] and cyanobacteria *Synechococcus elongatus* PCC 7942 (about 120 mg/L) [22, 23] as well as pure 2,3-BDO stereoisomer production in *E. coli* [24–28] has also been reported. However, most of these strains are aerobic or classified as risk group 2 microorganisms, which are not suitable for commercial production due to EPA biosafety regulations. The cost of aeration at large scale is also widely considered a problem for economic production. Moreover, most native microorganisms produce mixtures of three 2,3-BDO stereoisomers and, thus, metabolic engineering efforts are needed for production of pure stereoisomers [29–33].

*Zymomonas mobilis* is well known for both its high specific glucose uptake rate and rapid catabolism, and is engineered to metabolize all major biomass sugars [34–38]. Improved *Z. mobilis* strains developed by DuPont and NREL have been used for commercial-scale cellulosic bioethanol production in DuPont’s cellulosic ethanol plant at Nevada, IA, which is currently licensed to China and Macedonia. A novel deacetylation and disc-refining (DDR) process, also known as deacetylation and mechanical refining (DMR), followed by enzymatic hydrolysis, has been shown to result in low toxicity, high concentration mixed sugar syrups that are capable of producing high product titers in the biological upgrading of these sugars [39, 40]. Superior fermentation performance using *Z. mobilis* for conversion of DDR or DMR sugar streams to ethanol has been demonstrated [41, 42]. In addition, different approaches have been applied to develop the robust *Z. mobilis* 8b strains to enhance ethanol productivity in the presence of pretreatment inhibitors. These approaches include classical chemical mutagenesis and adaptation, transposon mutagenesis, as well as the forward genetics approach that resulted in the development of various robust strains [36, 43–49], such as the hydrolysate-tolerant mutant 8b-OEL22C (22C) [44]. To broaden the antibiotics availability for further metabolic engineering of commercially relevant *Z. mobilis* strains, a new *Z. mobilis* strain, 9C, was generated from its parental strain, 8b [37], with both tetracycline and chloramphenicol antibiotics markers removed. We found that the performance of strain 9C in different sugar sources is same as that of 8b (Unpublished data).

Since the genome sequence and functional re-annotation were reported [50, 51], a substantial library of systems biology data as well as several metabolic modeling studies have been accumulated in recent years to better understand the inhibitor tolerance mechanisms of *Z. mobilis* [52–66]. These inhibitors include the end-product ethanol and toxic compounds from pretreated biomass, such as acetate, furfural, and soluble phenolic compounds. This information laid a solid foundation for future systems biology studies and provided data for omics-guided metabolic engineering practices in *Z. mobilis*.

This work is focused on developing a novel process for anaerobic 2,3-BDO production from biomass derived mixed C5/C6 sugar streams. We aim to take advantage of *Z. mobilis*’ capabilities for rapid and efficient utilization of biomass-derived mixed sugar streams and redirect the carbon flux from ethanol to heterologous 2,3-BDO production to avoid the problem of mixed stereoisomers in native strains for pure stereoisomer production. Once sufficiently understood, we will work to develop novel pathways for advanced biological upgrading of sugars to high carbon efficiency intermediates amenable to separations and catalytic upgrading to hydrocarbon fuels.

## Results

### Investigate 2,3-BDO toxicity to *Z. mobilis*

**Fig. 1:**
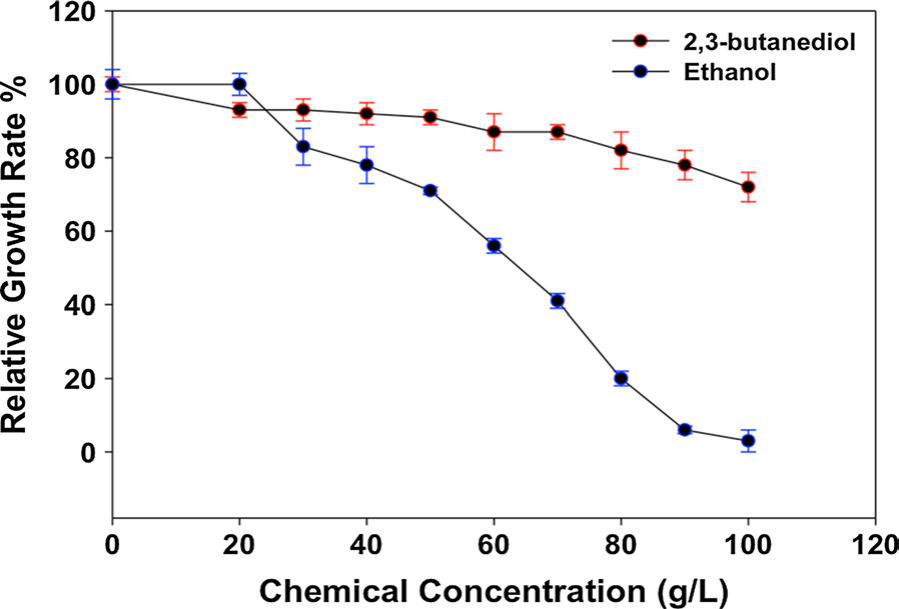
The impact of *meso*-2,3-BDO and ethanol supplementation on growth of *Z. mobilis* in RMG medium at 30 °C using Bioscreen C. Relative growth rate (%) is the percentage of growth rate with chemical supplementation compared to control without

First, we examined the feasibility of engineering *Z. mobilis* for high 2,3-BDO production by investigating the toxicity of 2,3-BDO to *Z. mobilis* in RMG2 medium at 30 °C using the Bioscreen C high-throughput growth measurement instrument. Our result showed that *Z. mobilis* tolerates higher concentration of 2,3-BDO than that of ethanol (Fig. 1). The growth rate decreased to 0.3 h^−1^ when about 80 g/L 2,3-BDO was supplemented to the growth medium, whereas less than 45 g/L ethanol was needed to slow the growth of *Z. mobilis* to similar growth rate. When 80 g/L ethanol was added into the medium, the growth rate of *Z. mobilis* was only about one-third of that observed for the same amount of 2,3-BDO added (Fig. 1). Even when the concentration of 2,3-BDO was increased to 100 g/L, the growth rate of *Z. mobilis* decreased to 0.25 h^−1^, but it was still more than half of the control without 2,3-BDO supplementation. The low toxicity of 2,3-BDO to *Z. mobilis* indicates that *Z. mobilis* has a strong potential for achieving high titers, rate, and yields for 2,3-BDO production.

### Construct a minimized vector for pathway engineering

To maximize the pathway engineering potential allowing for large plasmid constructions such as the three-gene 2,3-BDO pathway in this study, a minimized shuttle vector was constructed. The resultant plasmid, pEZ15Asp (pEZ), contains only the essential elements: origins of replication for both *E. coli* and *Z. mobilis*, an antibiotic marker of spectinomycin resistance gene *addA*, and multiple cloning sites and, therefore, significantly reduces its size when compared with the original plasmid (from 8 to 3 kb). Multiple cloning sites include restriction enzyme sites of *Eco*RI, *Xba*I, *Spe*I and *Pst*I for the Biobrick cloning strategy [67] enabling pathway engineering (see Table 1 and S1A for plasmid information and sequence).

**Table 1:** The source and components of the 2,3-BDO pathway constructed and the corresponding 2,3-BDO titer (g/L) after 24-h post-inoculation in *Z. mobilis* 8b

Strain or plasmid	Characteristic(s)	Source or reference
Plasmids		
pEZ15Asp (pEZ)	Shuttle vector contains *Z. mobilis* origin and *E. coli* origin p15A; Sp^R^; Biobrick-compatible	This work
pEZ-GS6	pEZ containing construct GS6: *aldC* and *bdh* genes from *E. cloacae* driven by its native promoter [PEc_(ALDC-BDH)]	This work
pEZ-GS2	pEZ containing construct GS2: codon-optimized *aldC* and *bdh* genes from *E. cloacae* driven by *Pgap* promoter [GS2: Pgap_(ALDC-BDH)	This work
pEZ-BC3	pEZ containing construct BC3: codon-optimized *aldC* and *bdh* genes from *B. licheniformis* driven by *Pgap* and *Peno* promoter respectively [(Pgap_ALDC)-(Peno_BDH)]	This work
pEZ-BC4	pEZ containing construct BC4: *alS* gene from *E. cloacae* driven by its native promoter (PEc_ALSecwt) and GS6 construct [(PEc_ALSecwt)-GS6]	This work
pEZ-BC5	pEZ containing construct BC5: codon-optimized *alS* gene from *E. cloacae* driven by *Peno* (Peno_ALSecopt) and GS2 construct [(Peno_ALSecopt)-GS2]	This work
pEZ-BC9	pEZ containing construct BC9: codon-optimized *alS* gene from *B. licheniformis* driven by inducible promoter *Ptet* (Ptet_BlAls) and GS2 construct [(Ptet-BlAls)-GS2]	This work
pEZ-BC10	pEZ containing construct BC10: codon-optimized *alS* gene from *B. subtilis* driven by inducible promoter *Ptet* (Ptet-BsAls1) and GS2 construct [(Ptet-BsAls1)-GS2]	This work
pEZ-BC11	pEZ containing construct BC11: 2nd version of codon-optimized *alS* gene from *B. subtilis* driven by inducible promoter *Ptet* (Ptet-BsAlss) and GS2 construct [(Ptet-BsAls2)-GS2]	This work
Bacterial strains		
*E. coli* C2925	*ara*-*14 leuB6 fhuA31 lacY1 tsx78 glnV44 galK2 galT22 mcrA dcm*-*6 hisG4 rfbD1 R(zgb210::Tn10)* Tc^S^ *endA1 rspL136* (Str^R^) *dam13::Tn9* (Cm^R^) *xylA*-*5 mtl*-*1 thi*-*1 mcrB1 hsdR2*	NEB
8b	Engineered *Z. mobilis* subsp. ZM4 strain for xylose utilization, Tc^R^Cm^R^	[37]
9C	8b derivative with tetracycline and chloramphenicol antibiotics markers cured, Tc^S^Cm^S^	Lab stock
22C	8b derivate with enhanced corn stover hydrolysate tolerance, Tc^R^Cm^R^	[44]
8b-pEZ	*Z. mobilis* 8b containing control plasmid pEZ	This work
8b-GS2	*Z. mobilis* 8b containing plasmid pEZ-GS2	This work
8b-GS6	*Z. mobilis* 8b containing plasmid pEZ-GS6	This work
8b-BC3	*Z. mobilis* 8b containing plasmid pEZ-BC3	This work
8b-BC4	*Z. mobilis* 8b containing plasmid pEZ-BC4	This work
8b-BC5	*Z. mobilis* 8b containing plasmid pEZ-BC5	This work
22C-pEZ	*Z. mobilis* 22C containing control plasmid pEZ	This work
22C-BC5	*Z. mobilis* 22C containing plasmid pEZ-BC5	This work
9C-BC5	*Z. mobilis* 9C containing plasmid pEZ-BC5	This work
9C-BC9	*Z. mobilis* 9C containing plasmid pEZ-BC9	This work
9C-BC10	*Z. mobilis* 9C containing plasmid pEZ-BC10	This work
9C-BC11	*Z. mobilis* 9C containing plasmid pEZ-BC11	This work

Culture condition: 40 mL RMG8 in 125-mL flask, 33 °C, 120 rpm

*EC* *E. cloacea*; *BL* *B. lincheniformis*. *Sp* ^*R*^ spectinomycin resistance; *Tc* ^*R*^ tetracycline resistance; *Cm* ^*R*^ chloramphenicol resistance; *Tc* ^*S*^ tetracycline sensitive; *Cm* ^*R*^ chloramphenicol sensitive

### Identify and select 2,3-BDO pathway genes for heterologous 2,3-BDO pathway construction

Literature related to 2,3-BDO production was reviewed, which includes the analysis of native 2,3-BDO biosynthesis pathways in addition to the expression of heterologous enzymes for 2,3-BDO production [1, 2, 5, 10, 12, 13, 15, 16, 19, 20, 23, 25, 27, 68, 69]. The 2,3-BDO pathways from *Bacillus licheniformis* and *Enterobacter cloacae* have been successfully expressed in *E. coli* [13, 16, 30, 33, 70, 71], suggesting the feasibility of 2,3-BDO production in *Z. mobilis*. The gene combination resulting in high 2,3-BDO yields and experimental methods reported in these papers laid a solid foundation for our work. We then compiled a list of 2,3-BDO pathway genes and the promising source microorganisms. After eliminating pathogenic organisms from our list, we chose two bacterial species, *B. licheniformis* and *E. cloacae,* for the source of our three-gene 2,3-BDO biosynthesis pathway reported here.

The sequences (nucleotide and protein) of three genes (*als, aldC,* and *bdh*) were extracted and BLASTed against the *Z. mobilis* genome including our recently updated native plasmid sequences. BLAST results indicate that Bdh only has homologs with low similarity in *Z. mobilis*; therefore, heterologous *bdh* genes were included in our pathway engineering strategy. In addition, the reaction that Bdh catalyzes is a reversible reaction, with some enzymes preferring NADPH as a cofactor. Bdh enzymes preferring the reduction reaction from acetoin to butanediol using NADH were selected for minimizing redox imbalance and for achieving high 2,3-BDO yield.

Additional BLAST results for the other two enzymes required for 2,3-BDO production indicate that *Z. mobilis* possesses three homologs to Als, of which ZMO1139/ZMO1140 (IlvB/C) may form a complex contributing to amino acid biosynthesis. The homolog ZMO0687 has a high similarity to the catabolic enzyme, Als. The high similarity between Als, IlvB (ZMO1139), and Pdc (ZMO1360) based on protein sequence alignment indicates that there could potentially be competition among them for substrate (pyruvate) binding and utilization. Additionally, since Pdc in *Z. mobilis* is required for ethanol production, providing energy, and maintaining redox balance for robust cellular growth, strategies to shift the carbon flux toward 2,3-BDO production are required. This can be accomplished by inhibiting *pdc* gene expression and Pdc enzyme activity or by enhancing the performance of the heterologous Als enzyme.

Gene expression patterns of the Als homologous gene in *Z. mobilis* were subsequently examined using public and in-house transcriptomic datasets of microarray and next-generation sequencing (NGS)-based RNA-Seq. The result indicated that the *als* gene homologs are abundantly expressed under different conditions, and that ZMO0687 is also differentially expressed in cells grown from media containing different carbon sources (e.g., downregulated in xylose medium compared to glucose). Furthermore, ZMO1141, which catalyzes the formation of (R)-2,3-dihydroxy-3-methylbutanoate from α-acetolactate (hereafter referred to as acetolactate) for valine, isoleucine, and leucine biosynthesis, has an abundant transcript level of average log_2_-based microarray intensity result of 13.78 compared to 14.69 for *pdc* (Additional file 1: Table S1B); therefore, the acetolactate decarboxylase (AldC) to be engineered should have a strong affinity for the substrate acetolactate to compete with ZMO1141, thus ensuring carbon flux toward acetoin and 2,3-BDO.

### Construct heterologous 2,3-BDO pathway in *Z. mobilis* and optimize flask fermentation conditions

**Fig. 2:**
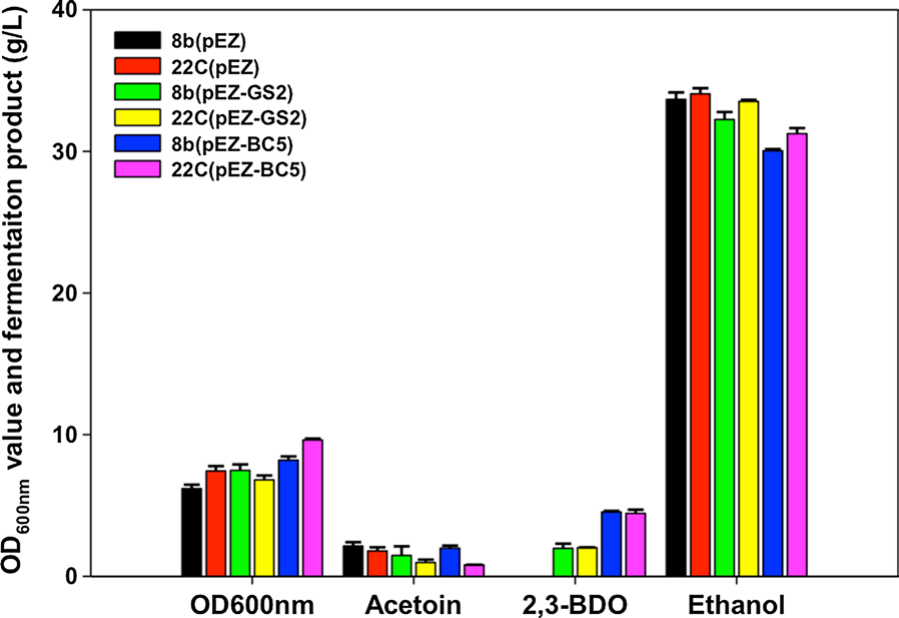
Biomass (OD_600 nm_), acetoin, 2,3-BDO, and ethanol titers after 24-h post-inoculation in either a *Z. mobilis* 8b and 22C background host with control plasmid pEZ15Asp (pEZ), or two-gene construct pEZ-GS2, or the three-gene construct pEZ-BC5 at 33 °C, 120 rpm with 40 mL RMG8 in 125-mL flask

The gene expression cassettes containing the sequences of three codon-optimized 2,3-BDO pathway genes (*als*, *aldC*, and *bdh*) from *B. licheniformis* and *E. cloacae* as well as wild-type version from *E. cloacae* were then designed and synthesized (Table 2). As discussed above, *Z. mobilis* has three *als* gene homologs, but does not have the *aldC* and *bdh* genes needed for 2,3-BDO production. Additionally, since *Z. mobilis* Pdc is a very abundant and efficient enzyme with low Km (<0.31 mM) and high *k*_cat_ (>100 1/s), a strong *als* gene may be needed to divert carbon flux from ethanol production to 2,3-BDO. We, therefore, constructed several pathways with either two genes (*aldC* and *bdh*) or three genes (*als*, *aldC,* and *bdh*). These genes were assembled using the Biobrick-based metabolic pathway engineering strategy and cloned into the minimized shuttle vector pEZ (Table 1). The results indicated that the three-gene pathways produced more 2,3-BDO than did the two-gene pathways (Table 2; Fig. 2).

**Table 2:** The source and components of the 2,3-BDO pathway constructed and the corresponding 2,3-BDO titer (g/L) after 24-h post-inoculation in *Z. mobilis* 8b

Strain	Plasmid	Promoter	Species	Enzymes	Codon optimization	BDO (g/L)
8b-GS6	pEZ-GS6	Wild-type	*E. cloacea*	AldC, Bdh	No	1.42
8b-GS2	pEZ-GS2	Pgap	*E. cloacea*	AldC, Bdh	Yes	3.69
8b-BC3	pEZ-BC3	Pgap, Peno	*B. lincheniformis*	AldC, Bdh	Yes	2.88
8b-BC4	pEZ-BC4	Wild-type	*E. cloacea*	Als, AldC, Bdh	No	1.9
8b-BC5	pEZ-BC5	Peno, Pgap	*E. cloacea*	Als, AldC, Bdh	Yes	5.0

Culture condition: 40 mL RMG8 in 125-mL flask, 33 °C, 120 rpm

*EC* *E. cloacea*; *BL* *B. licheniformis*

Three constructs (vector control pEZ, two-gene pathway plasmid pEZ-GS2, and three-gene pathway plasmid pEZ-BC5) were further compared in two strain backgrounds of 8b or 22C (Table 1). The introduction of heterologous *als* gene significantly increased 2,3-BDO production by shifting carbon flow away from ethanol production (Fig. 2).

Clearly 2,3-BDO production could be significantly enhanced if the native ethanol production pathway was impeded by knocking out key ethanol production genes of pyruvate decarboxylase (*pdc*) and alcohol dehydrogenase (*adh*), which has been demonstrated in *S. cerevisiae* that 2,3-BDO production increased in Pdc-deficient mutants [5, 7–10]. We have attempted to knock out or knock down the *pdc* gene to divert carbon to 2,3-BDO production without success, a result consistent with previous reports that *pdc* is essential for native *Z. mobilis* and cannot be completely knocked out. Furthermore, our efforts testing *pdc* knockout in 2,3-BDO production strains, such as 9C-BC5 and 9C-BC11, were also not successful. We will continue to work on blocking the route from pyruvate to ethanol to maximize the potential 2,3-BDO titer with balanced redox using similar strategies as reported in yeast [5, 6, 72–74].

**Fig. 3:**
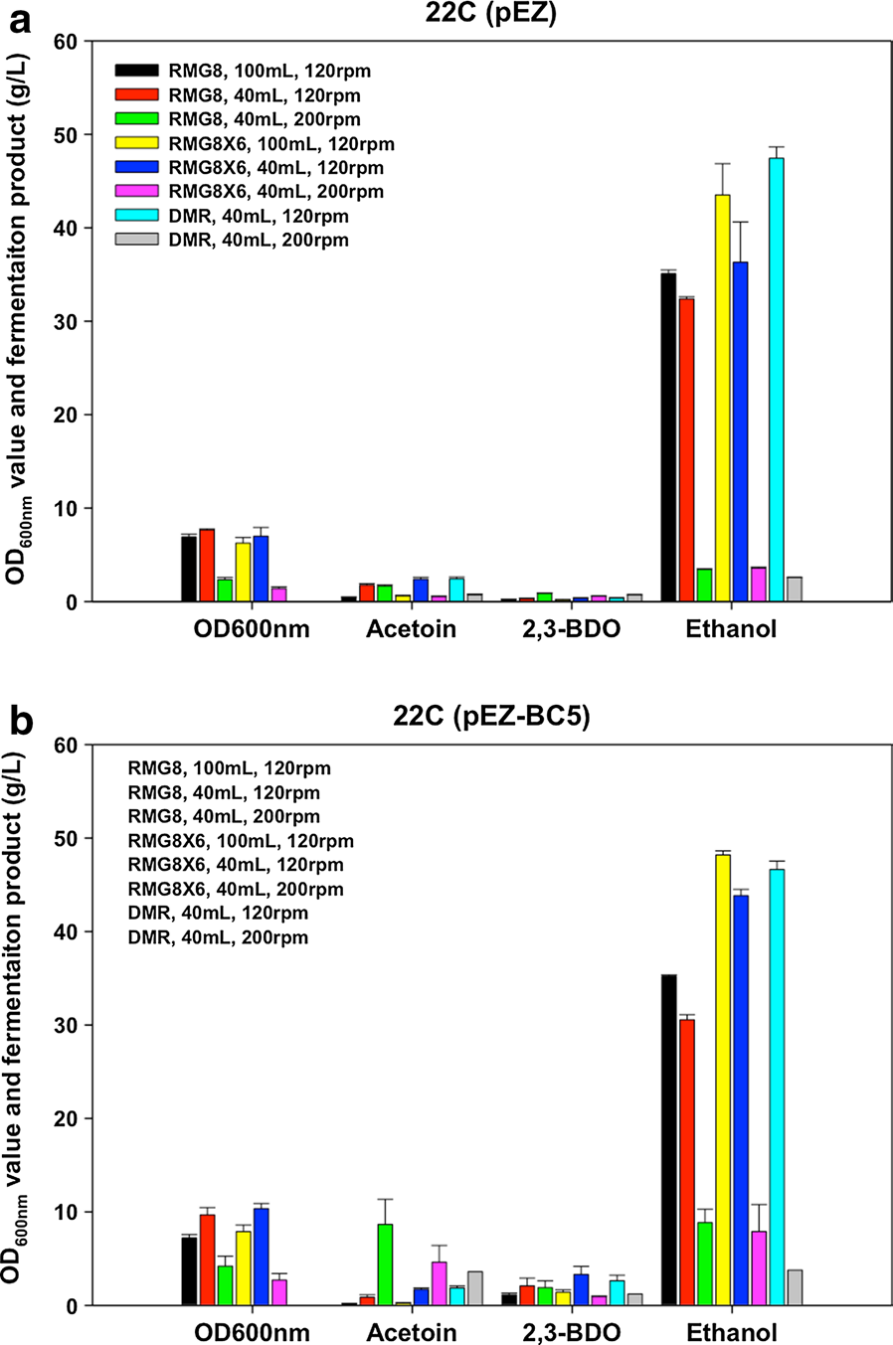
Biomass (OD_600 nm_), acetoin, 2,3-BDO, and ethanol titers after 24-h post-inoculation under different fermentation conditions which varied shaking speed (120 or 200 rpm), volume (100 or 40 mL in 125-mL flask), and media (RMG8, RMG8X6, and DMR) for control strain 22C(pEZ) (**a**) and three-gene pathway construct *Z. mobilis* 22C-BC5 (**b**) at 33 °C

To optimize the 2,3-BDO production, different flask fermentation conditions were first investigated using the three-gene pathway construct in the 22C background (22C-BC5) and using the empty vector pEZ as a negative control (Table 1; Fig. 3a). Results indicate that 2,3-BDO can be produced using mixed sugar conditions (glucose, or glucose and xylose), as well as from DMR-pretreated biomass (Fig. 3b). Growth conditions that varied the shaking speed and medium volume affecting oxygen dispersion impacted acetoin and 2,3-BDO production (Fig. 3b). For example, acetoin titers rose above 10 g/L when the shaking speed was increased to 200 rpm with 40 mL medium in the 125-mL baffled flask (Fig. 3b).

### Optimize heterologous 2,3-BDO pathway to increase 2,3-BDO titer

Initial 2,3-BDO engineering efforts in introducing heterologous *aldC* and *bdh* genes into *Z. mobilis* resulted in the 2,3-BDO production of about 3.7 g/L, which confirms that both *aldC* and *bdh* genes are required for 2,3-BDO production in *Z. mobilis*. Furthermore, the addition of a heterologous *als* gene from *E. cloacae* increased 2,3-BDO titers to about 5.0 g/L (Table 2), which again upheld the assumption that an additional *als* gene may be needed to redirect carbon flux from ethanol production into 2,3-BDO to compete with the abundant and highly active *Z. mobilis* Pdc enzyme. Our data thus suggested that the bottleneck reactions to increase 2,3-BDO production could be acetolactate generation from pyruvate and 2,3-BDO production from acetoin. We hypothesize that stronger *als* and *bdh* genes, or genes encoding more active enzymes, will boost BDO production.

To ascertain whether other heterologous *als* genes with higher specific enzymatic activities than *E. cloacae* one as reported in *E. coli* [16] could improve carbon flux from ethanol production into 2,3-BDO, *als* genes from *B. licheniformis* (BlAls) and *B. subtilis* (BsAls) were synthesized and cloned. These included one codon-optimized BlAls gene and two versions of the BsAls genes (BsAls1 and BsAls2) with different codon optimization outputs using Genscript’s codon optimization algorithm. The attempt to incorporate the *als* gene from *B. lichenformis* (BlAls) under control of the strong *Peno* promoter along with the expression of AldC and Bdh using the *Z. mobilisPgap* strong promoter was unsuccessful. Besides the possibility of cell growth inhibition by the enzyme Als from *B. licheniformis* and *B. subtilis,* this could be due to the protein burden caused by strong heterologous pathway gene expression. About 50 % of the proteins in *Z. mobilis* are involved in the glycolysis pathway [75]. This view is supported by a previous report that overexpression of plasmid-encoded protein led to the reduction of both glycolytic flux and growth rate due to protein burden [76]. Similarly, previous work also suggested that protein burden could be the reason for the negative effect observed for the isopropyl β-d-thiogalactoside (IPTG) induction of 2,3-BDO biosynthesis in a recombinant *E. coli* strain [26].

To test this speculation and avoid the potential metabolic burden caused by strong heterologous gene expression, the strong *Peno* promoter was tested along with the *Ptet* inducible promoter for the construction of additional three-gene pathway constructs. These constructs also included a common *aldC* and *bdh* gene operon from *E. cloacae* driven by a strong promoter *Pgap* [Pgap-(EcALDC-BDH)]. In total, six new 3-gene constructs with different versions of *als* genes from *B. lincheniformis* or *B. subtilis* were generated (BsAls1, BsAls2, and BlAls that were either driven by the strong *Peno* promoter or by the inducible *Ptet* promoter).

These six constructs were then transformed into *Z. mobilis* 9C (an 8b derivative that is lacking tetracycline and chloramphenicol resistance markers). Our result is consistent with our hypothesis that the transformation efficiencies were very high for plasmid constructs with the inducible promoter *Ptet* without tetracycline induction, but no positive transformants were obtained for constructs using the strong *Peno* promoter. 2,3-BDO production of three strains containing the three-gene construct with *als* driven by *Ptet* (Table 1) was measured, and the 2,3-BDO titer increased from 5–6 to 10–13 g/L (Table 3). In addition, it is apparent that the carbon was diverted from ethanol to 2,3-BDO production, considering the strong negative correlation between ethanol and BDO titers 3 days post-inoculation with an R-squared value of 0.98 (Table 3).

**Table 3:** The source and components of the 2,3-BDO pathway constructed and the corresponding BDO titer 1 day post-inoculation in *Z. mobilis* 9C

Strain	Plasmid	Promoter	als source	Enzymes	Acetoin (g/L)	BDO (g/L)	Ethanol (g/L)
9C-BC9	pEZ-BC9	Ptet	*B. licheniformis*	Als, AldC, Bdh	1.6 ± 0.4	10.8 ± 1.1	26.2 ± 0.5
9C-BC10	pEZ-BC10	Ptet	*B. subtilis*	Als, AldC, Bdh	1.1 ± 0.5	10.2 ± 2.0	27.4 ± 1.6
9C-BC11	pEZ-BC11	Ptet	*B. subtilis*	Als, AldC, Bdh	0.7 ± 0.1	13.3 ± 0.7	24.9 ± 0.5

The promoter of *als* gene in these constructed is driven by inducible promoter Ptet. Culture condition: 40 mL RMG8 with 200 μg/mL spectinomycin in 125-mL flask, 33 °C, 120 rpm. Four replicates each without tetracycline induction

**Fig. 4:**
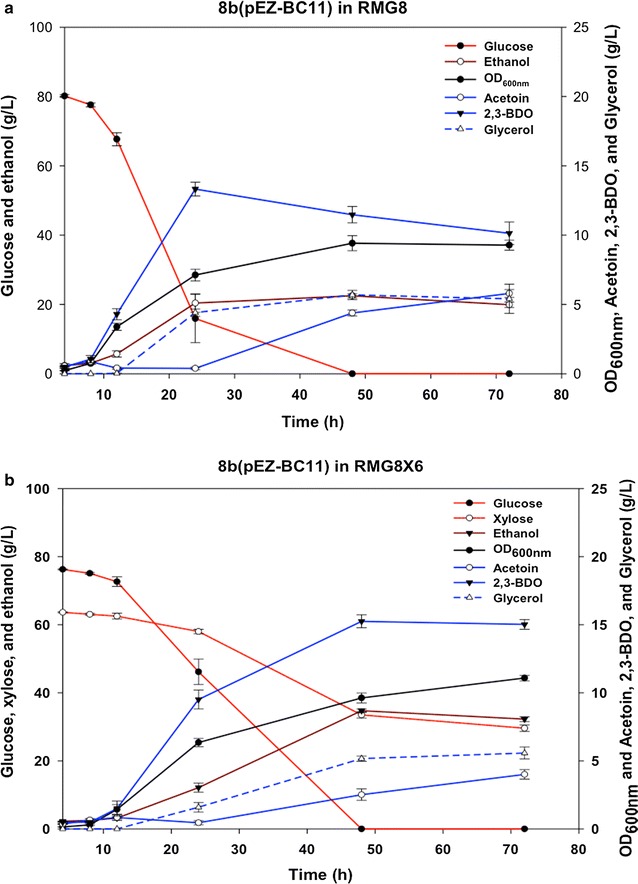
Biomass (OD_600 nm_), and concentrations of acetoin, 2,3-BDO, glycerol, ethanol as well as glucose and xylose for strain *Z. mobilis* 9C-BC11 grown in pure sugar of RMG8 (**a**) or mixed sugar of RMG8X6 (**b**). Culture condition: 40 mL RMG8 or RMG8X6 with 200 μg/mL spectinomycin in 125-mL flask, 33 °C, 120 rpm. Three replicates each without tetracycline induction

We further compared sugar consumption and 2,3-BDO production kinetics in glucose only or mixed sugars (glucose and xylose) with the new strain producing the highest 2,3-BDO titers, 9C-BC11 (Table 1). We found that this strain is stable, and 2,3-BDO titers were more than 13 g/L when grown in RMG8 or RMG8X6 media (Fig. 4). Notably, the addition of xylose delayed the sugar utilization of glucose and xylose; as well as cellular growth (Fig. 4). Furthermore, xylose was not completely utilized even 5 days after inoculation (data not shown). However, the concentration of 2,3-BDO in the mixed sugar fermentation remained level in stationary phase when compared to cultures grown in glucose only. In this case, levels of 2,3-BDO decreased after growth, possibly because of the reverse activity of Bdh converting 2,3-BDO back to acetoin, since decreasing 2,3-BDO titers was accompanied by in acetoin concentration increase (Fig. 4a).

### Investigate fermentation conditions on 2,3-BDO productions

**Fig. 5:**
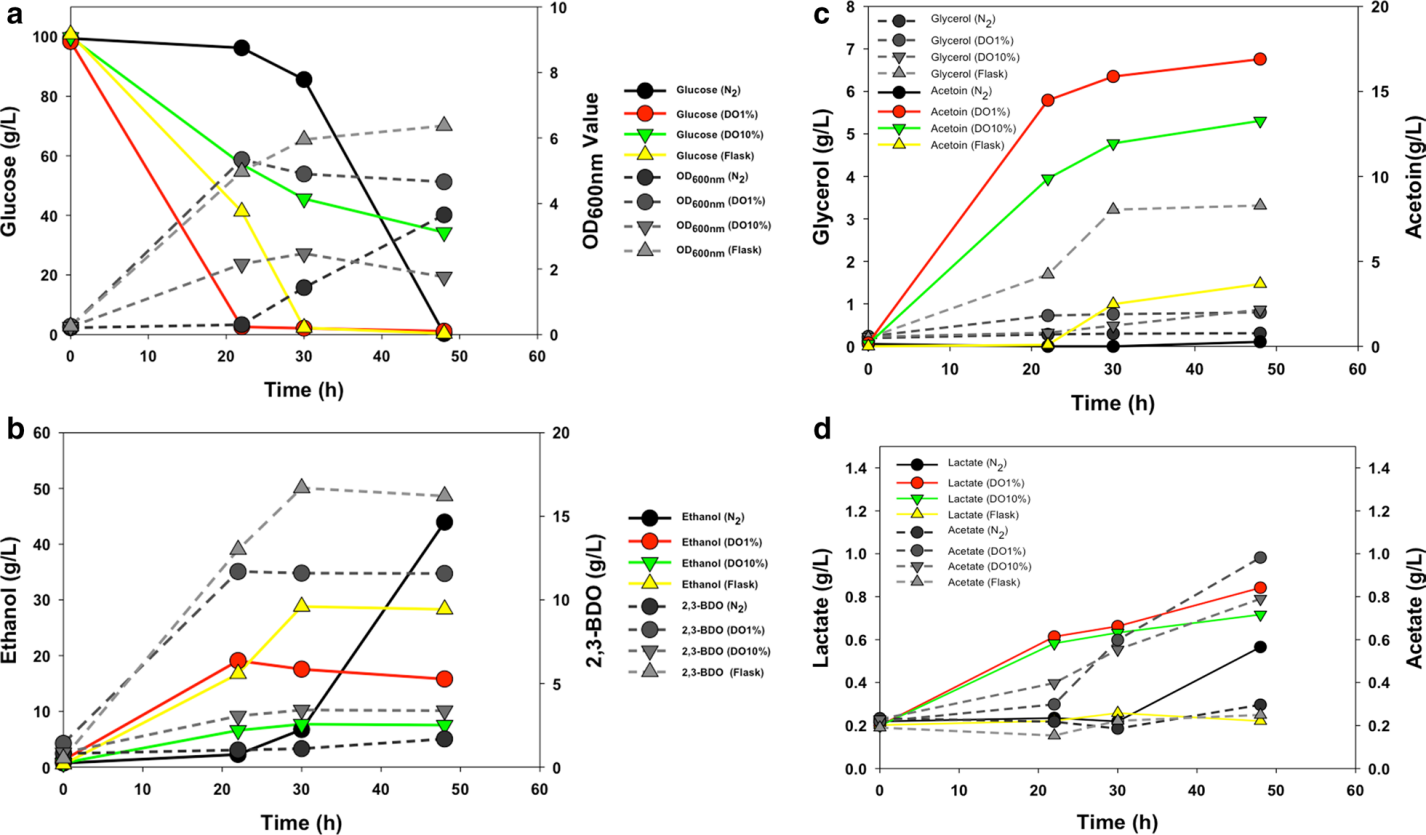
BioStat-Q plus fermentation profiles of glucose utilization and biomass (OD_600 nm_, **a**), 2,3-BDO and ethanol (**b**), acetoin and glycerol (**c**), acetate and lactate (**d**) of three-gene 2,3-BDO pathway construct *Z. mobilis* 9C-BC11 under different aeration conditions of N_2_ (0 % DO), 1, or 10 % DO (dissolved oxygen concentration) as well as shake flasks in RMG10 at pH 5.8 and 30 °C conditions 48-h post-inoculation

To further understand the impact of oxygen concentration on 2,3-BDO production and fermentation byproduct profiles as indicated by flask fermentation optimization (Fig. 3), Biostat-Q Plus fermenters (Sartorius Stedim North America Inc., Bohemia, NY) with oxygen control capability were used for our highest 2,3-BDO producing strain, 9C-BC11. As a control, shake flask fermentation was also conducted. Glucose is usually consumed within 20 h by the parental strain 9C under anaerobic conditions. Interestingly, the glucose utilization of *Z. mobilis* 9C-BC11 under anaerobic conditions with N_2_ purging was significantly reduced with only 3 g/L glucose consumed and no discernible cellular growth after 22 h post-inoculation. In contrast, more than half of the glucose had been used under shake flask conditions, and nearly all glucose was consumed upon reaching the highest OD_600 nm_ value of 5.34 under microaerophilic condition of 1 % dissolved oxygen (DO) (Fig. 5a). However, glucose utilization and cellular growth were significantly decreased when 10 % DO was supplied. At 48-h post-inoculation, there was still 34 g/L glucose remaining in the media, compared to other conditions tested where all glucose had been consumed (Fig. 5a), and the glucose level and OD_600 nm_ values remained relatively steady out to 120 h post-inoculation (Additional file 1: Table S1C).

Under anaerobic conditions with nitrogen sparging, nearly all glucose was ultimately converted into ethanol, producing only 1.68 g/L 2,3-BDO. Increasing oxygen deliveries from 0 to 1 % DO, or 10 % DO, decreased ethanol production dramatically (Fig. 5b), and is consistent with a previous report that aerobic growth reduces ethanol production [63]. Although both 2,3-BDO and acetoin levels increased under aerobic growth, titers of 2,3-BDO and acetoin were significantly higher in the 1 % DO culture than in the 10 % DO culture (Fig. 5b, c).

Besides the major end-products ethanol, acetoin, and 2,3-BDO, the production profiles of minor byproducts of acetate, lactate, and glycerol were also affected by the oxygen levels in these cultures. More acetate was produced under aerobic conditions resulting in less ethanol production as reported in the literature [63]. Furthermore, acetate production was significantly higher in the more aerobic conditions (1 or 10 % DO supplementation) than in the microaerophilic conditions using flask fermentation or N_2_-supplemented anaerobic fermentation (Fig. 5d). Additionally, more lactate was produced under aerobic conditions than in anaerobic condition as well (Fig. 5d). We had previously shown that the expression levels of the lactate dehydrogenase gene ZMO1237 were more abundant under aerobic conditions [63], and the expression of another d-lactate dehydrogenase gene, ZMO0256, was upregulated in the ethanol-treated cells during stationary phase [57]. The host strain, *Z. mobilis* 9C, used in this study, is a derivative of *Z. mobilis* 8b in which the ZMO1237 gene was inactivated [37]. Therefore, ZMO1237 may play the major role in lactate production under aerobic conditions. However, another lactate dehydrogenase gene, ZMO0256, could also be activated under aerobic conditions for lactate production, when ZMO1237 is deactivated, as shown in this study (Fig. 5d).

**Fig. 6:**
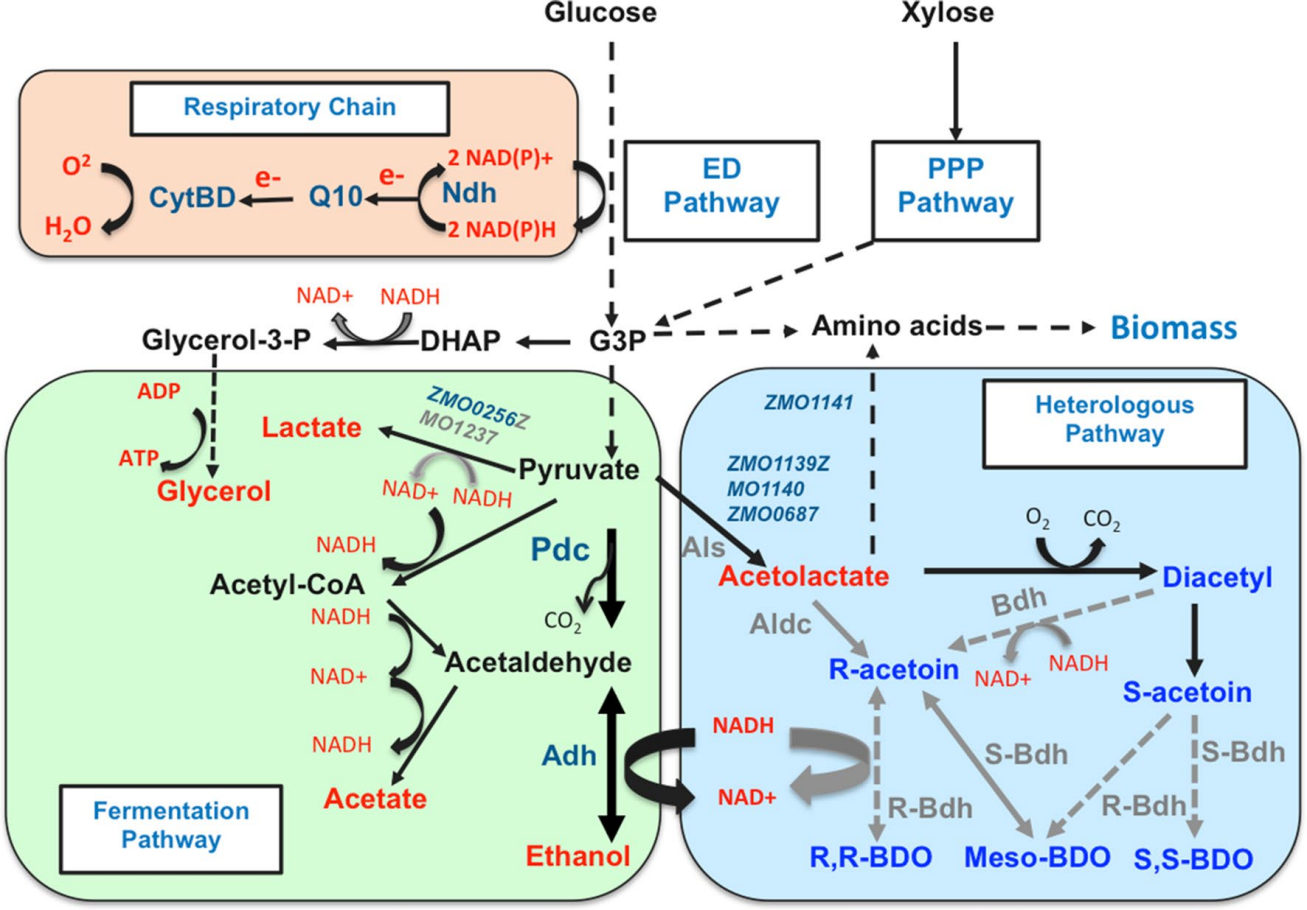
Impact of heterologous 2,3-BDO metabolic pathway engineering and oxygen levels on native central carbon metabolism in *Z. mobilis*. This figure shows native central carbon metabolism in *Z. mobilis* which includes ED (Entner–Doudoroff), PPP (pentose phosphate pathway), and fermentation pathways leading to the production of ethanol and other minor metabolites of glycerol, lactate, and acetate from carbon source of glucose and xylose. The heterologous 2,3-BDO biosynthesis pathway was integrated into *Z. mobilis* metabolic pathway, which contains three enzymes of acetolactate synthase (Als), acetolactate decarboxylase (AldC), and butanediol dehydrogenase (Bdh) for 2,3-BDO biosynthesis from pyruvate. Depending on the source of Bdh enzyme from different microorganisms, three 2,3-BOD stereoisomers of R,R-2,3-BDO, S,S-2,3-BDO, and *meso*-2,3-BDO could be produced. Reactions involved in redox cofactor (NADH/NAD+) regeneration were shown as well as the respiration chain enzymes and electron transfer in *Z. mobilis*. The interactions among these pathways can help understand the impact of host carbon and energetic metabolism as well as oxygen levels on heterologous 2,3-BDO metabolic pathway engineering in *Z. mobilis*

Intriguingly, despite the observation that more glycerol was produced under aerobic conditions (i.e., 1 or 10 % DO) than under anaerobic conditions with N_2_ purging, a higher amount of glycerol was produced in shake flask fermentations (see Fig. 5c). In *Z. mobilis*, only one NAD^+^ is regenerated from 2,3-BDO biosynthesis compared to two NAD^+^ from ethanol fermentation. The increase of glycerol production for in shake flasks may be explained by the utilization of the glycerol pathway to maintain redox balance (NAD^+^ feedback) while also provides ATP to sustain cellular growth (Fig. 6).

The highest 2,3-BDO yield (0.16 g/g glucose) was achieved under shake flask fermentations and the highest ethanol yield (0.44 g/g glucose) was obtained under anaerobic conditions using N_2_ purging (Additional file 1: Table S1C). As oxygen supplementation increased, acetoin yields also increased; however, these conditions also produced lower ethanol yields. In addition, the yields of all fermentation products (ethanol, lactate, glycerol, and acetate) were reduced with the increasing oxygen levels. For example, ethanol yields decreased from 0.46 g/g glucose under anaerobic conditions to 0.15 g/g glucose in the 10 % DO culture (Additional file 1: Table S1C). Conversely, the ratios of the heterologous products, acetoin and 2,3-BDO, to fermentation products increased from 0.04 g/g glucose under anaerobic conditions to 1.65 g/g glucose when 10 % DO was used, which reflects the higher acetoin yields and also correlated to lower ethanol yields under aerobic conditions (Additional file 1: Table S1C). We saw a similar result with shake flask fermentations. Higher acetoin production was accompanied by lower ethanol titer under more aerobic conditions (40 mL media in a 125-mL flask at 120 rpm) (Fig. 3b).

## Discussion

*Zymomonas mobilis* has attracted great attention due to its unique metabolic characteristics [49, 77–83]. Although it is missing two tricarboxylic acid cycle (TCA cycle) enzymes, malate dehydrogenase (Mdh) and 2-oxoglutarate dehydrogenase complex (Ogdh), as well as one key enzyme in the Embden–Meyerhof–Parnas (EMP) pathway (phosphofructokinase Pfk), *Z. mobilis* has outstanding fermentation performance consuming sugars at very high ethanol productivity rates and producing high ethanol titers through the Entner–Doudoroff (ED) pathway to provide energy for efficient growth. Glycolytic pathway enzymes are abundant in *Z. mobilis* which generate one ATP from each glucose molecule; compared to 2 ATPs generated from the EMP pathway and 38 ATPs from the TCA cycle. *Zymomonas mobilis* also has a respiration chain using oxygen as the terminal electron receptor under aerobic conditions. At least two branched electron transport systems have been proposed comprising NADH dehydrogenase (Ndh), coenzyme Q10, cytochrome bd, and O_2_, which has been confirmed by several studies [78, 81–87]. However, unlike other microorganisms, respiration in *Z. mobilis* is uncoupled to energetics and cellular growth with a function that has been suggested to maintain a low NADH/NAD^+^ ratio for efficient glycolysis and cellular growth [87]. Disturbance of the cellular NADH/NAD^+^ ratio due to external or internal inhibitors could lead to slow growth [48, 87]. A mutation of respiration chain genes, such as *ndh* mutation, or the supplementation of respiration chain inhibitors could result in better growth and higher ethanol titer under aerobic conditions [78, 81, 82, 84, 87] possibly due to more NADH becoming available for ethanol fermentation. However, excessive NADH would result in a high NADH/NAD^+^ ratio which would lead to inhibition of cellular growth [87]. Therefore, a well-balanced, low NADH/NAD^+^ ratio is key for efficient glycolysis and cellular growth in *Z. mobilis*.

Based on end-product data analysis for ethanol, acetoin, 2,3-BDO, lactate, acetate, and glycerol production, as well as cellular growth and glucose consumption profiles described above (Fig. 5), we propose the model below to explain the impact of host carbon and energetic metabolism as well as oxygen levels on heterologous 2,3-BDO metabolic pathway engineering in *Z. mobilis* (Fig. 6).

We hypothesize that during anaerobic conditions, there is insufficient NAD^+^ produced during ethanol fermentation to ensure efficient glucose catabolism in the engineered 2,3-BDO pathway in strain 9C-BC11 since pyruvate is being diverted away from ethanol production to 2,3-BDO. One acetolactate intermediate is synthesized from two pyruvate molecules by Als and then converted to acetoin and BDO by AldC and Bdh, respectively, oxidizing one NADH to generate one NAD^+^ as opposed to two NAD^+^ generated from ethanol fermentation. Moreover, the 2,3-BDO biosynthesis pathway may also compete for the substrate, acetolactate, with the branded-chain amino acids biosynthesis pathway for valine, alanine, and leucine. Taken together, the reduced NAD^+^ generated via the heterologous 2,3-BDO metabolic pathway and the competition for substrate with the amino acid biosynthesis pathway may increase the NADH/NAD^+^ ratio, which could then reduce the glycolysis efficiency and cellular growth resulting in a long lag phase (Fig. 5a). This lag could last until sufficient NAD^+^ could be generated to re-establish the optimal NADH/NAD^+^ ratio for efficient ethanol production.

Under 1 % DO culture conditions, we achieved the fastest glucose utilization and highest growth rates and the highest yields of 2,3-BDO and acetoin compared to anaerobic and 10 % DO conditions, suggesting that a more optimal redox balance is achieved under this condition. However, lower 2,3-BDO titers were realized compared to shake flask conditions. Clearly, the highest oxygen level tested (10 %) negatively impacted glucose utilization, cellular growth, and end-product yields (Fig. 5a, b, c), which may be due to an NADH/NAD^+^ ratio imbalance and thus inadequate NADH availability for 2,3-BDO biosynthesis and ethanol fermentation. Lactate dehydrogenase upregulated by oxygen may further exacerbate this situation by diverting needed NADH to lactate biosynthesis.

Under shake flask fermentation condition, more NAD^+^ may be generated from the acetoin to 2,3-BDO reaction, which could help maintain the redox balance. However, since the 2,3-BDO biosynthesis reaction is not as efficient as ethanol fermentation in *Z. mobilis* and NADH was not as efficiently recycled, we speculate that accumulation of more NADH is being diverted towards glycerol biosynthesis to regenerate NAD^+^ while also providing ATP for glycolysis and cellular growth. Since shake flask conditions achieved higher 2,3-BDO and ethanol yields with lower acetoin production than either 1 or 10 % DO fermentation conditions (Fig. 5b), we will further explore oxygenation levels and its impact on 2,3-BDO production and develop a strategy to balance redox to ensure maximum 2,3-BDO production under “anaerobic” or microaerophilic conditions.

The different physiological changes of a 2,3-BDO producer, therefore, could be contributed to the complex interplay between oxygen availability, the respiratory chain, oxygen and NADH cofactor requirement for heterologous 2,3-BDO biosynthesis and native glycolysis and ethanol fermentation pathways of *Z. mobilis.* In the presence of oxygen, *Z. mobilis* is able to oxidize NADH to NAD^+^ by the NADH oxidase Ndh in the respiratory chain. High levels of acetoin formation under these conditions suggested that NADH is oxidized to NAD^+^ by the NADH oxidase with high oxygen levels and will then not be available for the conversion of acetoin to 2,3-BDO by butanediol dehydrogenase (Bdh). Certainly, it is also possible that the activity of the Bdh is not optimal under these conditions and thus Bdh cannot compete with NADH oxidase. Further research will be needed to investigate the impact of oxygen, the fate of the NADH, and the Bdh enzymatic performance to optimize 2,3-BDO production and develop strategies for “anaerobic” production of 2,3-BDO in *Z. mobilis.*

## Conclusion

In summary, we have demonstrated the successful redirection of carbon flux from ethanol production in *Z. mobilis* to production of other hydrocarbon intermediates, such as 2,3-BDO, from glucose and xylose or mixed sugars from pretreated biomass hydrolysate. Our results indicate that all three genes of the heterologous 2,3-BDO biosynthesis pathway are essential for high 2,3-BDO production in *Z. mobilis*, and the bottleneck reactions are both the acetolactate generation from pyruvate and the 2,3-BDO production from acetoin. Pathway engineering to introduce Als with strong enzyme activity on pyruvate for acetolactate generation increased 2,3-BDO titer. In addition, our results reveal the impact of host cellular metabolism and the effect of oxygen levels on heterologous 2,3-BDO metabolic pathway engineering for 2,3-BDO production in *Z. mobilis* which can illuminate potential strategies for anaerobic 2,3-BDO production improvement. Furthermore, our study confirms the value of integrating bioinformatics analysis and systems biology data during metabolic engineering endeavors.

## Methods

### Bacterial strain and growth conditions

*Zymomonas mobilis* 8b was revived from frozen glycerol stocks for about 6–8 h in 10 mL RMG2 (20 g/L glucose, 10 g/L yeast extract, 2 g/L KH_2_PO_4_) at 33 °C. Other 8b derivatives used in this work are: 22C, a hydrolysate-tolerant mutant strain; 9C, an 8b derivative with both chloramphenicol and tetracycline resistance genes removed. The final spectinomycin concentration used for *Z. mobilis* is 200 and 50 μg/mL for *E. coli*.

### Growth curve measurement using Bioscreen C

Growth of *Z. mobilis* was monitored by Bioscreen C using the 420–580 nm filter (Growth Curves USA, NJ) as described previously without shaking [88, 89]. Three replicates were used for each condition. The seed cultures used for Bioscreen C were first revived from frozen glycerol stocks overnight in RMG2, then diluted 100-fold into fresh RMG2 until it reached exponential phase. The absorbance at OD_600 nm_ was adjusted to 3, then added 10-μL to each well containing 290-μL medium, such that the final OD_600 nm_ = 0.1. Procedures for measurement, recording of final cell densities, and calculations used to correct for non-linear response at high cell densities are previously reported [88, 89].

### Minimized shuttle vector construction and 2,3-BDO pathway assembly

A new minimized shuttle vector, pEZ15Asp, was designed and synthesized that includes origins of replication from both *E. coli* and *Z. mobilis*, antibiotics marker for the spectinomycin resistance gene, and multiple cloning sites for Biobrick-based pathway assembly [67].

Wild-type and codon-optimized versions of three 2,3-BDO biosynthesis pathway genes were synthesized by Genscript (NJ, USA). The genes were then assembled in different combinations using Biobrick-based approaches. Specifically, the minimized shuttle vector pEZ15Asp (pEZ) was digested with *Eco*RI-HF and *Pst*I-HF (Bio-Rad, CA), which was treated using the Rapid DNA Dephos and Ligation Kit (Roche, CA) for dephosphorylation and then quantified using Nanodrop and gel electrophoresis followed by gel purification. The insert(s) were also treated with restriction enzymes of *Eco*RI/*Spe*I or *Xba*I/*Pst*I and then quantified again using Nanodrop and gel electrophoresis followed by gel purification. The insert and vector were ligated using the fast-ligation kit (NEB, CA) with a molecular ratio of 3:1 for insert to vector. The ligation product (2-μL) was used for transformation into NEB C2925 competent cells. The transformants were confirmed by colony PCR using the primers of pBAD-GFP_F/R (5′TCACCAGCTCACCGTCTTTC3′ and 5′CCTGATGAATGCTCATCCGG3′) to confirm the insert size. The gene-specific primers were used to confirm that the targeted genes were cloned into the vector. Colonies showing the expected PCR bands were inoculated into LB broth supplemented with 50 μg/mL spectinomycin overnight and the plasmids were extracted and confirmed by PCR, restriction digestion, and Sanger sequencing.

### Electroporation transformation and 2,3-BDO strain selection

*Zymomonasmobilis* or *Escherichia coli* cells were transformed with plasmids by electroporation (Bio-Rad Gene Pulser, 0.1-cm gap cuvettes, 1.6 kV, 200 ohms, 25 μF). Electrocompetent *Z. mobilis* cells were prepared by centrifuging cells from cultures that had reached an OD_600 nm_ of 0.4–0.6. Cell pellets were washed once in ice-cold sterile water, re-centrifuged, and washed again in 10 % glycerol. These pellets were resuspended in 10 % glycerol at a concentration approximately 1000-fold higher than the starting culture. Competent cells were stored at −80 °C as small aliquots for later use. Transformants of *E. coli* or *Z. mobilis* were selected on LB or RMG agar plates, respectively, containing appropriate antibiotics. Due to the presence of restriction/modification systems in *Z. mobilis* [90] that can decrease transformation efficiency, all plasmids were built in and isolated from a methylation-deficient *E. coli* strain, C2925 (NEB, MA), for efficient transformation into *Z. mobilis* 8b or its derivatives.

For single colony isolation, transformants grown on the selective plates containing spectinomycin were further streaked on RMG plates with spectinomycin at a final concentration of 200 μg/mL (RMGSp). Following isolation, these colonies were then used for colony PCR to confirm the introduction of plasmids with correct pathway genes using the primers pBAD-GFP_F/R to check the insert size and gene-specific primers. Colonies with expected PCR bands pattern were selected and inoculated into RMGSp for preservation and further flask evaluation.

### Lignocellulosic biomass sugar hydrolysate

Biomass sugar hydrolysates were prepared using DMR corn stover. The details of DMR pretreatment process were described elsewhere [42]. In brief, corn stover (INL LOT#6, harvested in Hurley County, SD) was knife milled to pass through a 19 mm (3/4-in.) rejection screen followed by dilute alkaline (NaOH at 0.1 M) deacetylation at 80 °C in a 1900-L paddle mixer to remove most of the acetyl groups and some of the lignin. The deacetylated corn stover was disc refined at a specific refining energy of approximately 100 kW/ODMT in a pilot scale Sprout 401 (36-in.) disc refiner provided by Andritz, Inc., Springfield, OH. A secondary milling was applied to the deacetylated and disc refined corn stover substrates at 100kWh/ODMT using a planetary type Szego mill to further improve digestibility. The DMR-pretreated corn stover was then hydrolyzed using cellulases from Novozyme as reported previously [41]. The hydrolysis was conducted at 15 % total solids, pH 5.2, 50 °C and completed in 5 days.

### Shake flasks and Biostat-Q fermentations

Seed cultures of *Z. mobilis* strains harvested at exponential phase were inoculated into 125-mL shake flasks containing 40-mL media of RMG8 (RM with 80 g/L glucose) or RMG8X6 (RM with 80 g/L glucose and 60 g/L xylose) to a starting OD_600 nm_ of 0.1. The medium was supplemented with spectinomycin at a final concentration of 200 μg/mL. The temperature was maintained at 30 or 33 °C with a shaking speed of 120 rpm.

For BiosStat-Q Plus fermentations, strains were revived from frozen stock on RMG5 (50 g/L glucose) in 50 mL baffled flasks containing 10 mL of media, and incubated overnight at 30 °C in a shaking incubator at 180 rpm. The revived and grown culture was used to start the seed culture for fermentation. The seed cultures were then prepared in 125 mL shake flasks containing 40 mL RMG8 (80 g/L glucose) using revived cultures and inoculated at an initial OD_600 nm_ of 0.1. Seed flasks were incubated at 30 °C overnight in a shaking incubator at 180 rpm. Fermentations to evaluate the strains for BDO production were carried out in BioStat-Q plus fermenters with a 300-mL working volume of RMG with higher glucose concentration of 100 g/L (RMG10) to investigate whether higher carbon source could increase 2,3-BDO titer. The media were supplemented with spectinomycin at the final concentration of 200 μg/mL. The fermenters were inoculated from an overnight grown seed culture with an initial OD_600 nm_ value of 0.1. The fermenter operated at 30 °C, 300 rpm, and controlled at pH 5.8 with 4 N KOH.

### High-performance liquid chromatography (HPLC)

The dry weight of cells at the end of fermentation was determined by centrifuging 10 mL of broth and washing the cells with double deionized H_2_O. The washed cells were placed in pre-weighed aluminum dishes and dried overnight at an 85 °C incubator. The correlation between dry cell weight and OD_600 nm_ value was then calculated and used to estimate the dry cell weight in other time points.

Samples from the shake flasks or fermenters were taken at various time points. The samples were diluted for OD_600 nm_ measurement. In addition, samples were filtered through a 0.2-μm syringe filter into HPLC vials. Concentrations of glucose, xylose, 2,3-BDO, acetoin, xylitol, ethanol, HMF, furfural, lactic acid, glycerol, and acetic acid were determined from filtered sample supernatants by Agilent 1100 series HPLC (Agilent, CA) utilizing a BioRad Aminex HPX-87H organic acids column and Cation H^+^ guard cartridge (Bio-Rad, CA) operating at 65 °C. A refractive index detector was used for compound detection. Dilute sulfuric acid (0.01 N) was used as the isocratic mobile phase at a flow rate of 0.6 mL/min, following published procedures [41]. Sugar utilization, 2,3-BDO, acetoin, and ethanol titers were calculated based on the HPLC and dry cell weight data.

## Additional file

10.1186/s13068-016-0606-y Sequence information for constructs and major plasmids (1A) used in this work, systems biology datasets used to evaluate *Z. mobilis* genes that may potentially affect lactate and heterologous 2,3-BDO production (1B), as well as fermentation data and yield at different conditions (1C).

## Abbreviations

2,3-BDO: 2,3-butanediol
Als: acetolactate synthase
AldC: acetolactate decarboxylase
Bdh: butanediol dehydrogenase
Pdc: pyruvate decarboxylase
Adh: alcohol dehydrogenase
Mdh: malate dehydrogenase
Ogdh: 2-oxoglutarate dehydrogenase complex
Ndh: NADH dehydrogenase
TCA cycle: tricarboxylic acid cycle
EMP: Embden–Meyerhof–Parnas pathway
ED: Entner–Doudoroff pathway
DDR: deacetylation and disc-refining
DMR: deacetylation and mechanical refining
NGS: next-generation sequencing
HPLC: high-performance liquid chromatography
